# Diagnostic Errors in Trauma Care

**Published:** 1992-08

**Authors:** G. Hughes

**Affiliations:** Consultant in A&E Medicine Bristol Royal Infirmary


### DIAGNOSTIC ERRORS IN TRAUMA CARE

### by H. R. Guly

Clinical Press Limited 1992, 200 pp. £12.50

Any doctor who has worked in an A&E Department will recall the relative ease with which clinical and radiological errors can occur; indeed all grades of medical staff are at risk. The medical defence organisations have been aware of this problem for many years and examples of A&E errors are highlighted in their annual reports.

This excellent pocket size paperback written by an experienced A&E Consultant provides a timely analysis of the problem.

The first 5 chapters discuss various aspects of diagnostic errors in general terms; the subsequent chapters go through each body region or part of a region in more detail, highlighting common and less common errors made and the reasons why. The last 2 chapters provide wise advice on what to do when a mistake is realised and how the situation can be handled.

The text throughout is a model of clear lucid writing and thought, although the clarity of the radiographs reproduced is less than satisfactory.

I would recommend this book to all doctors, of what ever grade, involved in the assessment of patients with injuries, including A&E doctors, General Practitioners and Orthopaedic Surgeons. Even senior and distinguished members of the medical profession will find many useful nuggets of information and wisdom in this excellent book.

It should be part of any A&E or Fracture Clinic library and could also be used to form the framework for a series of tutorials and teaching sessions.

Reviewed by G. Hughes
Consultant in A&E Medicine
Bristol Royal Infirmary

